# Oral Curcumin–Thioketal–Inulin Conjugate Micelles against Radiation–Induced Enteritis

**DOI:** 10.3390/antiox13040417

**Published:** 2024-03-29

**Authors:** Jintao Shen, Wencheng Jiao, Bochuan Yuan, Hua Xie, Ziyuan Chen, Meng Wei, Yingbao Sun, Yanping Wu, Feng Zhang, Zhangyu Li, Xu Jin, Lina Du, Yiguang Jin

**Affiliations:** 1 Beijing Institute of Radiation Medicine, Beijing 100850, China; 2 Department of Anesthesiology, Cancer Hospital Chinese Academy of Medical Sciences, Beijing 100191, China; 3 Department of Anesthesiology, Beijing Tiantan Hospital, Capital Medical University, Beijing 100070, China

**Keywords:** curcumin, micelles, radioprotection, radiation enteritis

## Abstract

Radiation–induced enteritis is an unavoidable complication associated with pelvic tumor radiotherapy, significantly influencing the prognosis of cancer patients. The limited availability of commercial gastrointestinal radioprotectors in clinical settings poses a substantial challenge in preventing radiation enteritis. Despite the inherent radioprotective characteristics of Cur in vitro, its poor solubility in water, instability, and low bioavailability lead to inferior therapeutic effects in vivo. Herein, we developed novel ROS-responsive micelles (CTI) from inulin and curcumin, aimed at mitigating radiation enteritis. CTI micelles had excellent solubility and stability. Importantly, CTI improved the cytotoxicity and bioavailability of curcumin, thereby showing enhanced effectiveness in neutralizing ROS induced by radiation, safeguarding against DNA damage, and reducing radiation-induced cellular mortality. Moreover, in a radiation enteritis mice model, CTI not only alleviated severe radiation-induced intestinal injury but also improved redox-related indicators and reduced inflammatory cytokine expression. Furthermore, CTI effectively increased gut microbiota abundance and maintained gut homeostasis. In conclusion, CTI could be a promising candidate for the clinical management of radiation enteritis. Our study provides a new perspective for radioprotection using natural antioxidants.

## 1. Introduction

Radiotherapy is a prevalent treatment modality for pelvic malignancies, including cervical, anal, rectal, and prostate tumors [1]. Despite advancements in three-dimensional conformal and intensity-modulated radiotherapy, inadvertent exposure of nearby healthy organs to radiation can lead to radiation-induced diseases [2]. The intestine, the second most radiation-sensitive organ in the body, is particularly susceptible to damage during pelvic radiation due to its location within the abdominal cavity [3]. Notably, over 75% of patients undergoing pelvic radiotherapy develop radiation enteritis, which significantly impairs their quality of life and may pose life-threatening risks [4]. The generation of reactive oxygen species (ROS) by radiation is a key contributor to radiotoxicity, causing DNA damage, cell death, and cytokine overexpression, culminating in severe intestinal damage [5,6]. Hence, antioxidant therapy represents a promising approach for treating radiation-induced enteritis.

Amifostine, developed based on its anti-ROS mechanism, is the sole radioprotector approved by the US Food and Drug Administration for the treatment of radiation-induced diseases. However, the particularly short half-life of amifostine results in a narrow therapeutic window, requiring high dosages for efficacy [7]. In addition, amifostine has systemic toxicity and can cause complications such as orthostatic hypotension even at low doses, thereby limiting its high-dose use [8]. Hence, the pursuit of radioprotective agents with enhanced antioxidant activity and reduced toxicity has become an essential objective in the medical field.

Natural polyphenols have attracted much attention in the radioprotective field for their potent ROS-scavenging ability [9,10,11,12,13]. Curcumin (Cur), a natural polyphenolic compound extracted from turmeric (*Curcuma longa*), is known for its various pharmacological functions, including robust anti-inflammatory and antioxidative properties [14,15,16]. Meanwhile, numerous studies have demonstrated the robust radiation-protective ability of Cur in vitro [17]. However, the clinical efficacy of Cur is limited due to its poor water solubility, instability, low bioavailability, and rapid clearance from the body [18].

A common approach to address the aforementioned issues is to nano-encapsulate Cur [19,20,21]. Over the years, biocompatible and water-soluble polymers, notably polysaccharides, have garnered significant attention in developing nanosized polymer-drug conjugates [22,23]. The grafting of Cur onto polysaccharides has been demonstrated to enhance their solubility, permeability, and stability, thereby improving biological activity. [24,25]. Hence, it is logical to hypothesize that employing a polysaccharide-based carrier could enhance the radioprotective efficacy of Cur. However, this assumption remains unexplored to date.

Among a variety of polysaccharides, inulin (Inu) has garnered our interest. Inu, a naturally occurring and biologically safe fructan, is classified as a dietary fiber due to its notable properties, including biodegradability, renewability, nontoxicity [26,27]. Inu can also be used as a wall material in the encapsulation of drugs and bioactive compounds [28,29,30].

**Scheme 1:**
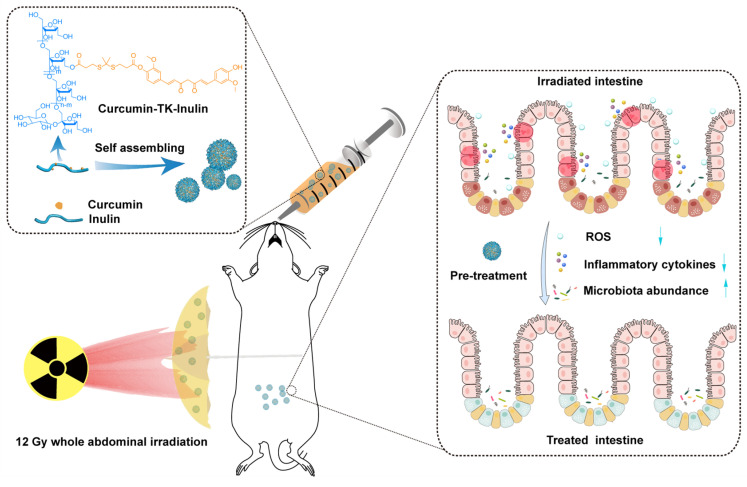
Oral curcumin–thioketal–inulin conjugate micelles against radiation-induced enteritis.

In this work, an amphiphilic conjugate, curcumin–thioketal–inulin (CTI), was designed and synthesized to fabricate an ROS-responsive micellar system to enhance the therapeutic efficacy of Cur in radiation-induced enteritis. Cur was conjugated with the backbone of Inu via a thiol-based ROS-responsive thioketal linker. The successful synthesis of CTI was confirmed through various characterization techniques, including FT-IR, ^1^H NMR, etc. The particle size distribution, morphology, physiological stability, critical micelle concentration (CMC), stability, and in vitro drug release behaviors of CTI were thoroughly investigated. The radioprotection effects and mechanisms of CTI were deeply studied using irradiated cells and mice (Scheme 1). These experimental results suggest that CTI could be a promising candidate for the clinical management of radiation-induced enteritis.

## 2. Materials and Methods

### 2.1. Materials

Inulin from chicory (MW ≈ 5000 Da) was provided by Sigma-Aldrich (St. Louis, MO, USA), 3-mercaptopropionic acid, acetone, trifluoroacetic acid, curcumin, pyridine, acetic anhydride, ethyl acetate, 1-(3-dimethylaminopropyl)-3-ethylcarbodiimide hydrochloride (EDC), N-hydroxy-succinimide (NHS) and sodium sulfate (Na_2_SO_4_) were purchased from Beijing InnoChem Science and Technology Co., Ltd. (Beijing, China). Dulbecco’s modified Eagle’s medium (DMEM) and fetal bovine serum (FBS) were purchased from Gibco Life Technologies (Grand Island, NY, USA). Cell Counting Kit 8 (CCK-8) and live/dead cell staining kit were purchased from Beijing Solarbio Science and Technology Co., Ltd. (Beijing, China). The enzyme-linked immunosorbent assay (ELISA) kits of interleukin 6 (IL-6), and tumor necrosis factor *α* (TNF-*α*) were purchased from Shanghai Mlbio Biotechnology Co., Ltd. (Shanghai, China). Deionized water was prepared using the Heal Force Super NW Water System (Shanghai Canrex Analytic Instrument Co., Ltd., Shanghai, China). All other reagents were of analytical grade without further purification.

### 2.2. Synthesis and Characterization of CTI Conjugate

The synthesis and characterization details of the CTI conjugate are provided in the Supplementary Materials.

### 2.3. Physicochemical Characterizations of CTI Micelles

Physicochemical characterizations including particle size distribution, morphology, physiological stability, critical micelle concentration (CMC), stability, and in vitro drug release behaviors are provided in the Supplementary Materials.

### 2.4. In Vitro Antioxidant Properties

The antioxidant properties of Inu, Cur, and CTI micelles were investigated by 2,2′-azinobis(3-ethylbenzothiazoline)-6-sulfonic acid (ABTS) radicals, 1,1-diphenyl-2-picrylhydrazyl (DPPH) radicals, and hydroxyl (OH•) radical-scavenging assays [31]. Each experiment was performed in triplicate.

ABTS radical-scavenging assays were accomplished via the overnight reaction between 2.45 mM potassium peroxydisulfate and 7 mM ABTS solution in the dark. The working solution with an absorbance of 0.70 ± 0.02 at 734 nm was obtained by dilution with anhydrous ethanol. Then, each concentration of samples of 50 μL was co-incubated with the working solution of 50 μL in the dark for 30 min. The optical density (OD) at 517 nm was measured, and the ABTS radical-eliminating effect was calculated with Equation (1):

EA (%) = (OD_control_ − OD_sample_)/OD_positive_ × 100% (1)

where EA denotes ABTS radicals eliminating effect, OD_control_ denotes the absorbance at 734 nm of the control group, and the absorbance of the sample groups are marked as OD_sample_.

To assess the scavenging effect of DPPH radicals, each concentration of samples of 50 μL was prepared in a 96 well-plate. Then, 50 µL DPPH solution (0.5 mM in ethanol) was added and incubated for 30 min in the dark. In the control group, 50 µL PBS was used instead of samples. The optical density (OD) at 517 nm was measured, and the eliminating effect of samples on DPPH radicals was calculated using Equation (2).

ED (%) = (OD_control_ − OD_sample_)/OD_positive_ × 100% (2)

where ED denotes the DPPH-elimination rate and ED_sample_ and ED_control_ the absorbance of the samples and the control groups at 517 nm, respectively.

An electron spin resonance (ESR) assay was conducted to determine the elimination ability of CTI on OH• radicals. Briefly, a reaction solution containing 0.4 mM ferrous sulfate, 400 mmol 5,5-dimethyl-1-pyrroline-N-oxide (DMPO), deionized water (pH 4.5), and 10 µL 4% H_2_O_2_ was quickly prepared and loaded in a quartz capillary. After a reaction of 30 s, the ESR spectrum was recorded between 330 mT and 340 mT using a Bruker A300 EPR spectrometer. For the experimental groups, deionized water was replaced by different concentrations of samples.

### 2.5. Cytotoxicity Assay

IEC-6 cells seeded in a complete DMEM containing 10% fresh fetal bovine serum, 1% penicillin, and streptomycin and cultured in a 5% CO_2_, 37 °C incubator were used for cellular experiments.

The cytotoxicity of the Inu, Cur, and CTI micelles on IEC-6 cells was measured via the CCK-8 assay. Briefly, IEC-6 cells were seeded into a 96-well plate (5 × 10^3^ cells/well) and incubated overnight for adherence. Afterward, the medium was replaced with new media with a series of concentrations of samples. After culturing for 24 h, cells were washed twice with PBS, followed by incubation with CCK-8 solutions for 2 h, and then the optical density was measured at 450 nm with a microplate reader (Spark, Tecan, Männedorf, Switzerland). The same volume of DMEM was used as the control group. The wells without cells were added with the same volumes of CCK-8 solution as the blank group. The data are expressed as means ± SD (n = 3). The cell viability (CV) of samples was calculated with Equation (3):

CV (%) = (OD_sample_ − OD_blank_)/(OD_control_ − OD_blank_) × 100% (3)

where OD_sample_, OD_control_, and OD_blank_ are the absorbance values of the experimental, the control, and the blank groups, respectively.

### 2.6. In Vitro Cellular Uptake

IEC-6 cells were inoculated in a 12-well plate at a density of 3 × 10^4^ cells/well. After 24 h, cells were cultivated with 10 µg/mL of Cur and 100 µg/mL of CTI micelles for 12 h at 37 °C, respectively. After incubation, the cells were washed twice with PBS and stained using DAPI. The viability and morphology of cells were observed by fluorescence microscopy (Olympus, Tokyo, Japan). Cellular uptake levels were also quantitatively analyzed by flow cytometry. Data were analyzed using FlowJo V10 software (Becton-Dickinsom, Franklin Lakes, NJ, USA).

### 2.7. In Vitro γ-ray Damage Evaluation

A CCK-8 assay was performed to assess the damage caused by *γ*-ray to IEC6 cells. In brief, cells were inoculated in a 96-well plate at a density of 5 × 10^3^ cells/well and incubated overnight for adherence. Afterward, cells were treated by *γ*-ray irradiation at various doses (4, 8, 12 16, and 20 Gy, 1.5 Gy/min). After culturing for 48 h, cells were washed twice with PBS, followed by incubation with CCK-8 solutions for 2 h (37 °C, 5% CO_2_). Finally, a microplate reader (Spark, Tecan, Männedorf, Switzerland) was used to determine the absorbance at 450 nm.

### 2.8. In Vitro Radioprotection Assay

Briefly, IEC-6 cells were inoculated in a 96-well plate at a density of 5 × 10^3^ cells/well for 24 h. Following attachment, the cells were seeded with 10 µg/mL of Cur or 100 µg/mL of CTI micelles for 4 h. Subsequently, cells were exposed to a 12 Gy dose of *γ*-ray irradiation. After 48 h, cells were washed twice with PBS, followed by the addition of CCK-8 solution and incubation for another 1 h. Finally, the optical density was determined with a microplate reader at 450 nm.

For the calcein–PI assay, the medium was replaced with 500 μL of DMEM containing 2 mM calcein AM and 4 mM propidium iodide (PI). A calcein–PI solution (1 μL/mL calcein–1 μL/mL PI) was added and cultured at 37 °C for 30 min. Subsequently, the cells were extensively washed with PBS to eliminate any surplus fluorescence markers. Cellular viability and morphology were then examined using fluorescence microscopy (Olympus, Tokyo, Japan).

### 2.9. Apoptosis Assay

Apoptosis assay was carried out following the instructions of an Annexin V-FITC/PI Apoptosis Detection Kit (Yeasen, 40302ES, Shanghai, China). In short, IEC-6 cells were seeded in a 6-well plate at a density of 1 × 10^5^ cells/well for 24 h. After attachment, the cells were co-incubated with 10 µg/mL of Cur or 100 µg/mL of CTI for 4 h prior to 12 Gy dose of *γ*-ray irradiation. Subsequently, cells were exposed to a 12 Gy dose of *γ*-ray irradiation. After 24 h, cells were collected and detected with a flow cytometer (BD II, BD Biosciences, Franklin Lakes, NJ, USA) after annexin V–FITC/PI staining.

### 2.10. Intracellular ROS-Scavenging Assay

The intracellular ROS level in IEC-6 cells in response to irradiation stimulation was evaluated according to a previous study [32]. Briefly, IEC-6 cells were seeded in a 12-well plate (5 × 10^4^ cells/well) and cultured in complete DMEM for 24 h. Then, cells were treated with 10 µg/mL of Cur or 100 µg/mL of CTI micelles for 4 h. Subsequently, cells were exposed to a 12 Gy dose of *γ*-ray irradiation. After 24 h, cells were stained with 10 μmol/L of DCFH-DA for 20 min in the dark at 37 °C. The fluorescence images of cells were observed by fluorescence microscopy. For the microplate reader assay, IEC-6 cells were digested and seeded into 96-well plates and their fluorescence intensity at 525 nm was assessed with a microplate reader at an excitation wavelength of 488 nm. The cells without irradiation were considered the control group.

### 2.11. Detection of γH2AX

Histone H2AX (*γ*H2AX) was used to investigate DNA double-strand breaks (DSBs). The IEC-6 cells were incubated with a density of 10 × 10^5^ cells per confocal microscope dish. After attachment, cells were treated with 10 µg/mL of Cur or 100 µg/mL of CTI micelles for 4 h. Then, 24 h after irradiation, the cells were fixed with 4% paraformaldehyde for 20 min and washed with PBS and treated with 0.2% Triton-X 100 for 15 min. After PBS washing, the cells were incubated with rabbit polyclonal *γ*H2AX (phospho S139) primary antibody (dilution 1:1000; G266; Dojindo, Tokyo, Japan) for 1 h. After discarding the primary antibody, the cells were gently washed with PBS three times and incubated with goat anti-rabbit secondary antibody (G266; Dojindo) at room temperature for 1 h. The nuclei were counterstained with DAPI (C0065, Solarbio, Beijing, China). Images of the cells were collected by fluorescence microscopy (Olympus, Tokyo, Japan).

### 2.12. Colony Formation Assay

IEC-6 cells were seeded in a 12-well plate at a density of 100 cells/well and cultivated for 24 h. Then, the cells were incubated with 10 µg/mL of Cur or 100 µg/mL of CTI micelles for 4 h, followed by 12 Gy of *γ*-ray irradiation. The medium was changed every two days. Seven days after irradiation, methanol was used to fix the cells, which were then stained with Giemsa for 10 min. Subsequently, the cells were washed with distilled water twice after discarding the dye solution. The number of colonies was calculated by ImageJ 1.6 software (US National Institutes of Health, Stapleton, NY, USA).

### 2.13. Radiation Enteritis Model

C57BL/6J mice (6 weeks, male, SPF, 20–22 g) were purchased from SPF Biotechnology Co., Ltd. (Beijing, China). All the animal experiments were approved by the Animal Care and Use Committee of the Academy of Military Medical Science (IACUC-DWZX-2022-834), in accordance with the Guidelines for the Care and Use of Laboratory Animals published by the National Research Council. The experimental protocols were performed in accordance with ARRIVE guidelines.

The mouse radiation enteritis model was established by ^60^Co *γ*-ray whole abdominal irradiation (WAI). Briefly, C57BL/6J mice were randomly divided into five groups (n = 10). Model, Cur, and CTI were administered orally with 300 μL of PBS, Cur (1.25 mg/mL), and CTI micelles (12.5 mg/mL) by gavage 1 h before WAI and 12 h after WAI, respectively; 150 mg/kg of amifostine was used as a positive control and administered intraperitoneally 30 min before WAI [33]. The mice were exposed to 12 Gy of *γ*-ray irradiation with a dose rate of 75.52 cGy/min. Lead plates were used to shield the mouse’s body except the abdomen during *γ*-ray irradiation.

### 2.14. Evaluation of WAI-Induced Intestinal Injury

Body weight, survival condition and vitality of mice were carefully observed for 14 days. The small intestinal tissues were gathered and fixed in 5% formaldehyde on day 3. The tissue sections were then stained using H&E and Ki67. All sections were examined with a microscope (BDS200-FL, Chongqing Optec Instrument Co., Ltd., Chongqing, China). Morphological analysis of the small intestines (including villus length and regenerate crypts) was conducted using ImageJ software (US National Institutes of Health, Stapleton, NY, USA).

### 2.15. Evaluation of Intestinal Permeability

Intestinal permeability was evaluated by an FITC–dextran assay. In brief, C57BL/6 mice were fasted for 12 h, but allowed water uptake. FITC–dextran (3–5 kDa, Sigma Aldrich) was administered orally at a dose of 600 mg/kg in 0.3 mL PBS on day 7 postirradiation. After gavage 4 h, serum was collected and transferred into black 96-well plates (Corning Incorporated, Corning, NY, USA). The fluorescence intensity of serum was measured using a fluorescence spectrophotometer (Spark, Tecan, Männedorf, Switzerland) with the excitation wavelength at 480 nm and the emission wavelength at 530 nm. Each experiment was performed in triplicate.

### 2.16. Determination of Inflammatory Cytokines and Redox-Related Indicators

The excised intestinal samples were homogenized (120 Hz, 4 min) with sterile saline to obtain 10% (*w*/*v*) homogenates. The tissue homogenates were centrifuged at 5000× *g*, 4 °C for 20 min. Supernatants were collected to quantitatively evaluate the levels of the inflammatory cytokines (TNF-*α*, IL-1*β* and IL-6) and redox-related indicators (MDA and SOD) in accordance with the instructions of the ELISA kits.

### 2.17. Behavioral Evaluation

The open field test (OFT) was used to evaluate the physical activity of mice [34]. All mice were placed in a quiet room to avoid environment effects. Tested mice were placed in the testing area of the OFT (Beijing Zhongshi Dichuang Technology Development Co., Ltd., Beijing, China) with a plastic chamber (50 cm × 50 cm × 50 cm) for 5 min. Total distance and dead time were recorded using a video camera connected to a computer with Labmaze V3.0 tracking system software.

### 2.18. Microbiome Analysis

Feces of mice were collected on day 7 postirradiation and sent to Suzhou Genewiz Biotechnology Co., Ltd., Suzhou, China for microbiome analysis. The forward primer (CCTACGGRRBGCASCAGKVRVGAAT) and the reverse primer (TACNVGGGTWTCTAATCC) were used to amplify the V3–V4 variable regions in 16s rDNA genes. Sequencing libraries were generated using a TruSeq DNA PCR Free Sample Preparation Kit (Illumina, San Diego, CA, USA) according to the instructions of the manufacturer. Similar sequences were coded into operational taxonomy units (OTUs) based on 97% sequence identity. Silva132/16s_bacteria were used in the genus/species classification database with a rank confidence level of 0.7. Chao1, Shannon and Simpson indices were calculated to assess the alpha diversity of each sample. Nonmetric multidimensional scaling (NMDS) and principal component analysis (PCA) were used to assess beta diversity.

### 2.19. Statistical Analysis

All data are expressed as means ± standard deviation (S.D.). Data were calculated statistically using SPSS 17.0 and GraphPad Prism 8 statistical analysis software. Independent Student *t*-tests were used for the comparison of two groups, while one-way analysis of variance (ANOVA) followed by Tukey’s test were used to identify the statistical differences between multiple groups. *p* values < 0.05 were considered statistically significant.

## 3. Results and Discussion

### 3.1. Characteristics of Cur–TK–Inu Conjugate

**Figure 1:**
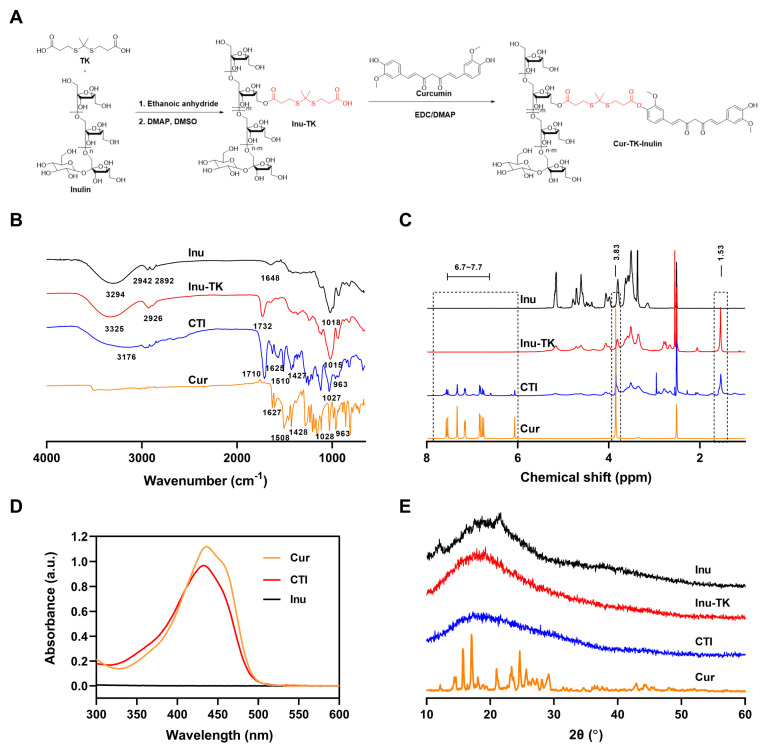
(**A**) Synthetic route and characteristics of the CTI conjugate. (**B**) FTIR spectra, (**C**) ^1^H NMR spectra, (**D**) UV-vis spectra, (**E**) XRD spectra of Cur, Inu, CTI, and Inu–TK conjugate.

The conjugation of hydrophobic drugs with hydrophilic polymers not only improves the aqueous solubility of the drugs but also reduces toxicity, overcomes drug resistance mechanisms, and improves overall therapeutic efficacy [35]. This study aimed to conjugate Cur with a hydrophilic Inu backbone through an ROS-sensitive linker—thioketal. For the synthesis of CTI, Inu was first modified to introduce carboxylic acid (-COOH) functionality, followed by conjugation with Cur via an esterification reaction (Figure 1A). The synthesis of carboxyl terminated thioketal (TK) was carried out as per previously reported literature [36], and its structure was identified by mass spectrometry and ^1^H NMR (Figures S1 and S2). Subsequently, carboxylated thioketal was catalyzed by acetic anhydride to form anhydride thioketal, which can easily further react with Inu to form Inu–TK.

The successful synthesis of the CTI conjugate was confirmed by FTIR spectra and ^1^H NMR spectra. In FTIR spectra, the primary signals of inulin appeared at 3294 cm^−1^ (stretching vibrations of O–H), 2942 cm^−1^ (stretching vibration of C–H), and 1018 cm^−1^ (stretching vibration of C–O and C–C). Compared with inulin, a novel sharp band appeared at 1732 cm^−1^ (C=O stretching of esters), which verified the successful synthesis of Inu–TK (Figure 1B). After the introduction of Cur, new peaks at 1510 cm^−1^, attributed to the keto CߝO groups of Cur, were observed. Furthermore, a significant reduction in the intensity of the 951 cm^−1^ peaks associated with mannuronic acid (M) was observed during the conjugation process. This finding indicates that the esterification of the carboxylic group occurred within the M block. As shown in Figure 1C, the characteristic peaks of Inu can be found at 3.3–4.2 ppm (fructose ring) and the emergence of peaks ascribed to methyl groups (1.53 ppm) verified the successful yield of Inu–TK. Then, in the ^1^H NMR spectrum of the CTI conjugate, characteristic correlation peaks of Cur aromatic ring protons were displayed at 6.59–7.56 ppm, indicating the successful coupling of Cur with Inu–TK.

The conjugation of Cur with Inu was additionally verified through UV-vis spectroscopy. The UV-vis spectra demonstrated that the Cur-CTI conjugate exhibited maximum absorption peaks at 435 nm and 432 nm, respectively. However, no significant absorption peaks were observed for Inu alone in the same wavelength range (Figure 1D). The content of Cur in the CTI conjugate was estimated to be approximately 10% by UV spectroscopy.

XRD patterns of Cur exhibited peaks at 12.16°, 15.7°, 17.06°, 20.98°, 23.34°, 24.64°, and 25.62°, indicating a high crystalline state (Figure 1E). The broad band of Inu indicates the overall amorphous state. In CTI, the peaks of Cur disappeared, suggesting its non-crystalline presence within the conjugate, thus further providing evidence of conjugation.

### 3.2. Characteristics of CTI Micelles

**Figure 2:**
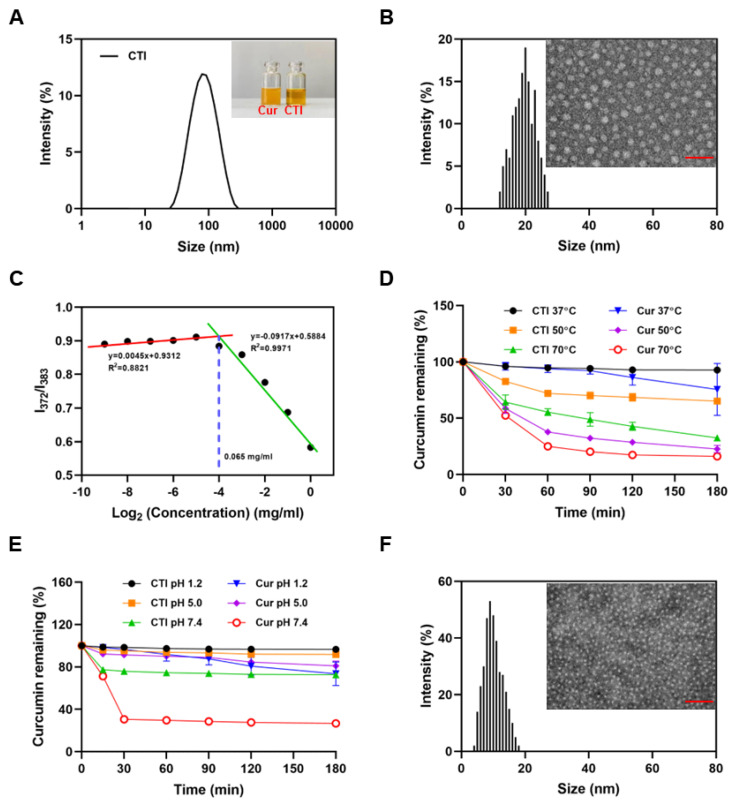
Characteristics of CTI micelles. (**A**) Particle size distribution of CTI micelles. Insert: digital photograph of Cur and CTI micelles, Scale bar: 100 nm. (**B**) TEM image and particle diameter distribution of CTI micelles. (**C**) CMC determination of CTI micelles. (**D**) Thermal stability and (**E**) pH stability of free curcumin and CTI. (**F**) TEM image and particle diameter distribution of CTI micelles after co-culture with ROS. Scale bar: 50 nm.

The average hydrodynamic diameter of CTI micelles, determined by dynamic light scattering, was 72.02 ± 2.27 nm with a PDI of 0.22 ± 0.02 (Figure 2A). The CTI micelle solution appeared homogeneous and nearly transparent. In contrast, the free Cur solution showed heterogeneity, with visible precipitation observed after 30 min (insert in Figure 2A). However, the TEM images and AFM images revealed that the size of the micelles was smaller than 30 nm (Figure 2B and Figure S3). This size reduction can be attributed to the shrinkage of the hydrophilic Inu chains in the corona region of the micelles during the drying process of TEM sample preparation.

The critical micelle concentration (CMC) is widely regarded as a conspicuous indication of the thermodynamic stability of polymer micelles [37]. Micelles can only be formed when the polymer concentration surpasses its CMC. The CMC value, determined by plotting the fluorescent intensity ratio I372:I383 against CTI concentrations, was found to be as low as 65 μg/mL. This suggests that CTI conjugates form nanomicelles at very low concentrations (Figure 2C). The water solubility of CTI was about 21.3 mg/mL, equivalent to 2.1 mg/mL of Cur (Figure S4). Compared with the solubility of free Cur (0.39 ± 0.05 μg/mL), the formation of CTI micelles increased solubility in water by approximately 5384-fold.

### 3.3. Stability of CTI Micelles

Cur degrades spontaneously in aqueous solutions, with changes in temperature and pH affecting its bioactivity. As shown in Figure 2D, the degradation rate of Cur accelerates with increasing temperature. After incubation for 3 h at 50 °C, only 13.8 ± 3.3% Cur in free Cur remained while the retention rate of Cur in CTI reached 59.9 ± 2.5%, which suggested a substantial enhancement in the thermal stability of Cur following the coupling process.

The considerable fluctuations in pH levels within the gastrointestinal tract have a profound impact on the bioavailability of Cur. Cur exhibits its highest stability within the pH range of 1–6; however, its stability is notably compromised when the pH surpasses 7 [38]. The free Cur content after 3 h of incubation decreased by 26.5 ± 11.1% at pH 1.2, 15.5 ± 4.5% at pH 5.0, and 73.2 ± 0.75% at pH 7.4, respectively. With identical treatment conditions, Cur in CTI exhibited reductions of 3.4 ± 0.3%, 8.2 ± 2.7%, and 27.4 ± 1.8% after 24 h of incubation at pH 1.2, pH 5.0, and pH 7.4 respectively (Figure 2E). The outcomes from stability experiments demonstrate that CTI micelles can efficiently mitigate Cur’s susceptibility to external conditions, thereby diminishing its degradation.

### 3.4. ROS-Sensitive Property of CTI Micelles

The thioketal bond present in TK is highly sensitive to ROS. TEM confirmed that the spherical nanostructure of CTI micelles collapsed and turned into small fragments under ROS conditions (Figure 2F). To simulate the Cur release from CTI micelles under physiological and inflammatory pathological conditions, in vitro drug release behavior was studied in PBS with or without 1 mM of H_2_O_2_. The release behaviors of Cur indicated that about 50% of drug was released within 48 h in the ROS environment while only about 18% drug was released in PBS (Figure S7). Rapid release of Cur in the ROS environment might be essential for the prevention of radiation enteritis.

### 3.5. In Vitro Antioxidant Ability of CTI

Ionizing radiation induces the overproduction of ROS in living organisms, resulting in sustained oxidative stress and cellular damage. Free radical scavenging has been demonstrated to be the most effective treatment for radiation-induced damage [39]. The scavenging abilities of ABTS (2,2′-azino-bis (3-ethylbenzothiazoline-6-sulfonic acid)) and DPPH (2,2-diphenyl-1-picrylhydrazyl) are commonly used as representative indices to evaluate the antioxidant efficiency.

**Figure 3:**
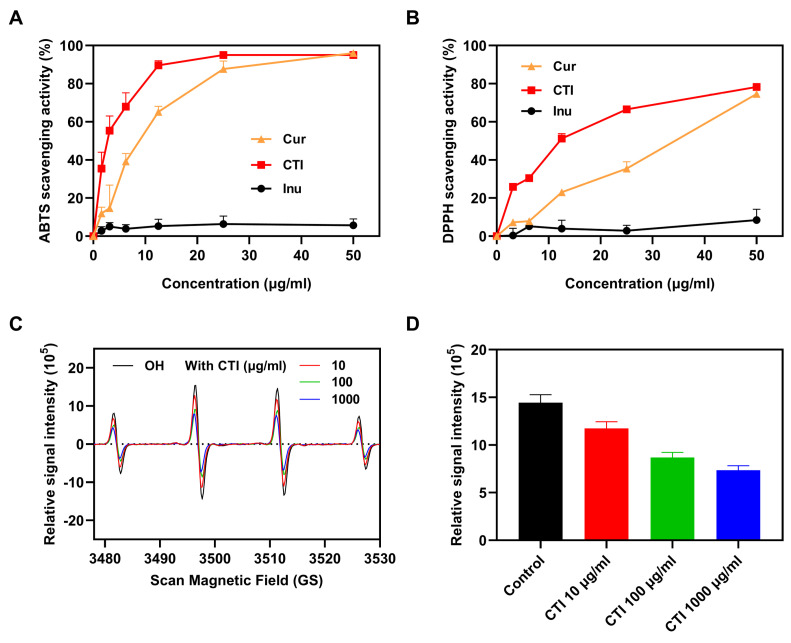
The antioxidant ability of CTI. The scavenging activities of (**A**) ABTS radicals and (**B**) DPPH radicals. (**C**) ESR spectrum of OH• incubation with the different concentrations of CTI micelles. For the control group, OH• radicals were incubated with PBS of the same volume. (**D**) ESR peak histogram of OH• (n = 4).

To investigate the impact of conjugation on the antioxidative capacity of Cur, we conducted a comparison of the ABTS and DPPH radical-scavenging activities among Cur, CTI micelles, and Inu alone. Both Cur and CTI exhibited enhanced ABTS and DPPH radical-scavenging activity, which increased with the concentration of Cur. Notably, Inu alone displayed no ABTS and DPPH radical-scavenging activity (Figure 3A,B). Since TK has been extensively reported on for its role in scavenging free radicals [40], we speculated the observed improvement results from the synergistic effect between TK and Cur. Hydroxyl (OH•) radicals are the most representative ROS produced by ionizing radiation. CTI micelles effectively scavenged oxygen free radicals (Figure 3C), with the scavenging rate of OH• free radicals increasing with CTI concentration (Figure 3D).

### 3.6. CTI Mitigates Cytotoxicity and Enhances Cell Uptake

**Figure 4:**
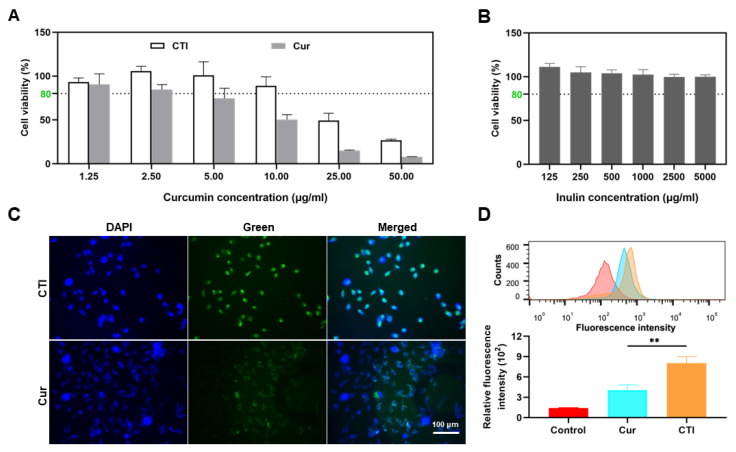
CTI mitigates cytotoxicity and enhances cell uptake. Cell viability of IEC-6 cells after incubation with different concentrations of (**A**) CTI and Cur and (**B**) Inu for 24 h. (**C**) Representative images of IEC-6 cells after incubation with Cur or CTI for 12 h. Scale bar: 100 μm. (**D**) Fluorescence intensity determined by flow cytometry of IEC-6 cells after incubation with CTI or Cur (** *p* < 0.01, mean ± SD, n = 3). For the control group, the cells were cultured with the complete medium only.

Cur is known for its antioxidant capabilities and its widespread use in radioprotection [41]. Thus, we initially investigated the physiological effects of CTI on cells. IEC-6, a typical small intestinal epithelial cell line, was utilized to evaluate the in vitro radioprotective efficacy of CTI [42]. First, the cytotoxicity of Cur, CTI, and Inu towards IEC-6 cells was assessed through a CCK-8 assay. The cytotoxicity of free Cur was significantly higher at high concentrations compared to an equivalent Cur concentration in the form of CTI, indicating a considerable reduction in cytotoxicity of CTI (Figure 4A). It was unsurprising to observe an improvement in the cytocompatibility of Cur upon conjugation with polysaccharides (Figure 4B).

Cellular uptake of Cur and CTI was evaluated by fluorescence microscopy. After 24 h of culture with CTI, green fluorescence was observed within the cells and co-localized with the blue fluorescence area (nuclei), indicating the internalization of the CTI via endocytosis (Figure 4C). Meanwhile, the intensity of green fluorescence in the control group, the Cur group, and the CTI group was quantitatively assessed via flow cytometry. Compared to the control group, the green fluorescence intensity in the CTI group significantly increased (*p* < 0.05), which suggests a higher cellular internalization of the radioprotective agents (Figure 4D).

### 3.7. CTI Protects Intestinal Cells from Radiation-Induced Damage

**Figure 5:**
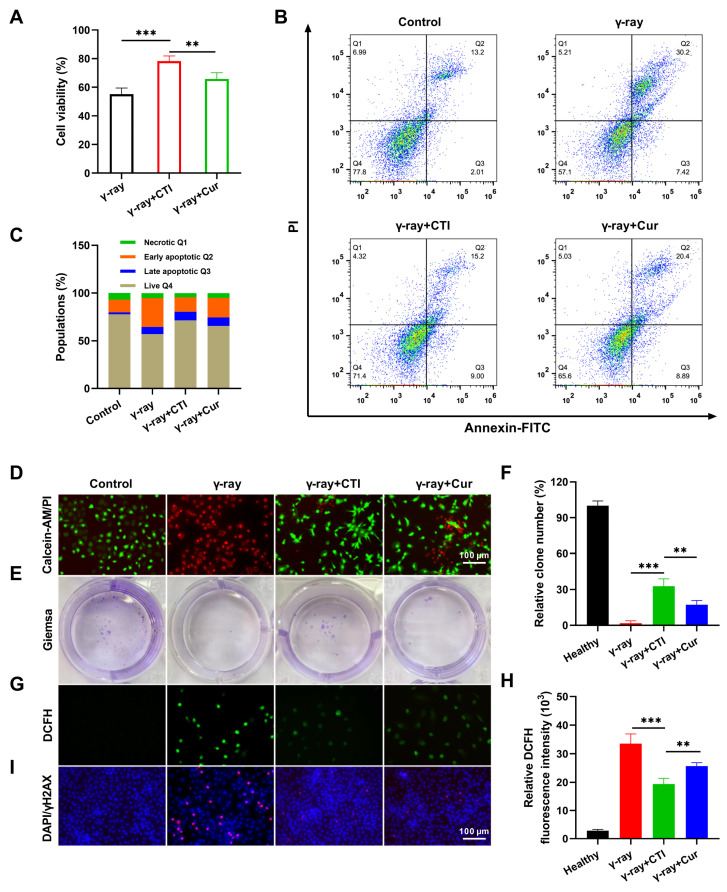
CTI protects intestinal cells from radiation-induced damage. (**A**) Cell viability of IEC-6 cells after different treatments. (**B**) Flow cytometry plots of IEC-6 cells after FITC–PI staining. (**C**) Population of cell apoptosis after radiation. (**D**) Calcein AM–PI dyeing of IEC-6 cells after different treatments. (**E**) Representative photographs of colony numbers with different treatments. (**F**) Quantitative analysis of the percentage of colony numbers after different treatments. (**G**) ROS levels were measured with a fluorescence microplate reader. (**H**) Intracellular ROS measurement of L929 cells by DCFH-DA staining (scale bars, 100 μm). (**I**) DNA double-strand damage in IEC-6 cells after different treatments by *γ*H2AX immunofluorescence assay. Data are presented as means ± SD, n = 3 (** *p* < 0.01, *** *p* < 0.001, n = 3).

A 12 Gy dose of *γ*-ray radiation was chosen for the subsequent in vitro radioprotective experiments (Figure S6). IEC-6 cell viability in the CTI group (78.2%) was significantly higher than in the model group (55.1%) (Figure 5A). Meanwhile, the flow cytometry results demonstrated the robust radiation protection effects of CTI and Cur with apoptosis rates of 24.2% and 29.29%, respectively, compared to the high apoptosis rate (37.62%) in the model group (Figure 5B,C). Moreover, calcein–AM/PI assays also revealed that *γ*-ray radiation induced significant cell death, while CTI and Cur significantly improved the viability of irradiated cells (Figure 5D). Additionally, a colony formation assay was conducted to assess the clonogenic survival of IEC-6 cells under different treatments. Compared to the nonirradiated group, the colony formation of cells in the model group was significantly reduced. In contrast, pretreatment with Cur or CTI prior to irradiation resulted in a significant increase in the number of colony formations (Figure 5E,F). Overall, these findings confirm the superior radioprotective capability of CTI towards normal IEC-6 cells compared to natural Cur.

Elevated levels of ROS directly induce DNA damage, leading to cellular injury [43]. Therefore, we assessed the capacity of CTI to mitigate ROS generation in IEC-6 cells exposed to *γ*-ray radiation. Following pretreatment with either free Cur or CTI, the green fluorescence intensity of intracellular ROS induced by *γ*-ray irradiation was significantly diminished compared to cells subjected to *γ*-ray treatment alone. The CTI group exhibited the lowest green fluorescence intensity. Fluorescence quantitative analysis results indicated that the CTI group exhibited the lowest fluorescence intensity among the irradiated groups (Figure 5G,H). Furthermore, we assessed the ability of CTI in mitigating radiation-induced DNA damage in IEC-6 cells through *γ*-H2AX immunofluorescence staining, a marker for double-stranded DNA breakage. After *γ*-ray irradiation, noticeable DNA damage in the model group was observed. However, in the Cur and CTI groups, a significant reduction in the presence of red fluorescent fluorescence was observed, indicating the diminished ROS expression by Cur and subsequent inhibition of the interaction between ROS and DNA (Figure 5I). The above results verified that CTI can effectively protect DNA from *γ*-ray-induced damage.

### 3.8. CTI Ameliorates Severe Radiation-Induced Intestinal Injury

**Figure 6:**
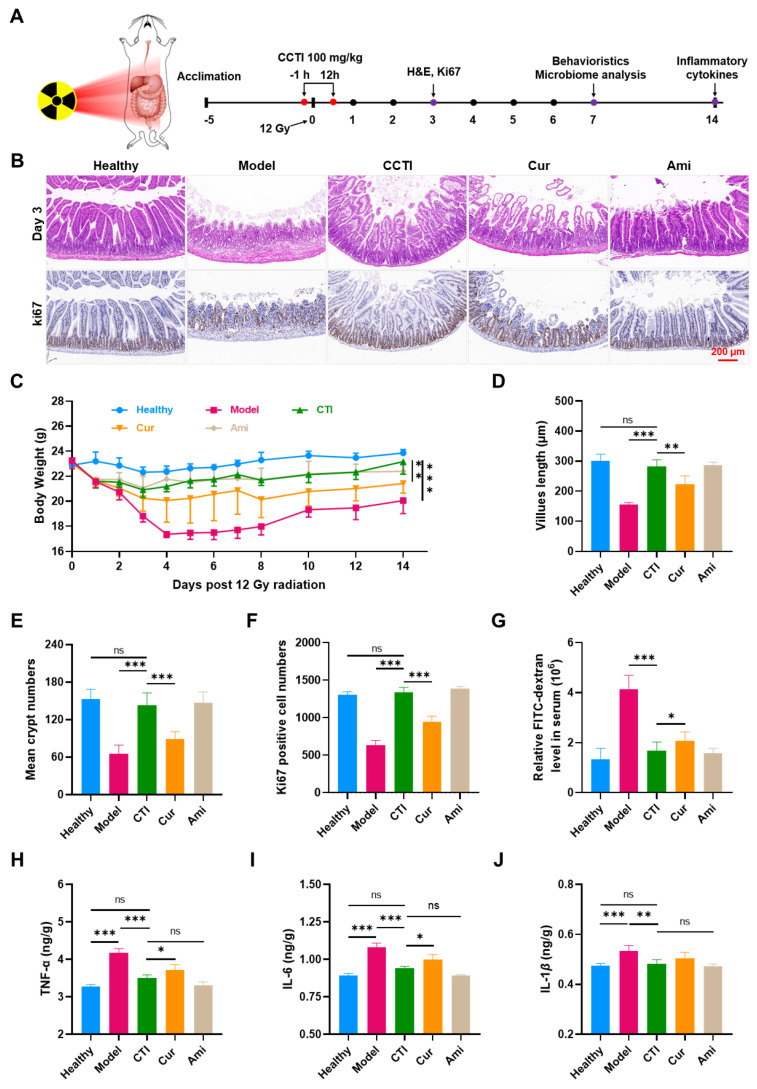
In vivo radioprotective effects of CTI. (**A**) Schematic illustration of CTI as a radioprotector against intestinal damage caused by 12 Gy dose of *γ*-ray irradiation. (**B**) H&E and Ki67 staining images of the intestinal sections on day 3 post irradiation. Scale bar = 100 μm. (**C**) Body weights of mice under *γ*-ray irradiation with different treatments. Error bars represent means ± SD (n = 6). Quantitative analysis of (**D**) villus length, (**E**) crypt numbers and (**F**) Ki67-positive cells on day 3. (**G**) Relative FITC-dextran levels in serum. Expression of (**H**) TNF-*α*, (**I**) IL-6 and (**J**) IL-1*β* in the small intestine tissues in different groups, Data are presented as means ± SD, n = 4. Student’s two-tailed *t*-test, ns: non-significance, * *p* < 0.05, ** *p* < 0.01, and *** *p* < 0.001.

With its rapidly proliferating and differentiating cells, the gastrointestinal system is highly susceptible to irradiation [44,45]. Irradiation affects the gastrointestinal system by specifically targeting the proliferating stem cells within the crypts, resulting in alterations to the histology and physiology of the intestine. Motivated by the promising radioprotective effects observed on intestinal cells, we thoroughly assessed the potential of CTI for radioprotection in normal intestinal tissues (Figure 6A).

Earlier research demonstrated that a 12 Gy dose of whole abdominal irradiation (WAI) was nonlethal and suitable for studying *γ*-ray-induced intestinal damage. Amifostine at a dose of 150 mg/kg body weight was chosen as a positive control to evaluate the therapeutic efficacy of CTI. After a period of 3 days, the small intestines were collected for subsequent pathological analysis. Compared to the healthy group, the crypt structure in the intestinal tissues of the model group was severely damaged. In contrast, mice pretreated with CTI and amifostine exhibited significantly less radiation-induced intestinal damage (Figure 6B,D). The administration of Cur improved the damage to the villi of mice compared to the *γ*-ray group. However, this improvement was limited compared to the CTI group and amifostine group, whose intestinal villi length was almost the same as the healthy group (Figure 6B,E). The small intestine is crucial for nutrient absorption and directly influences the body weight of mice. As shown in Figure 6C, the weight of mice subjected to *γ*-ray treatment exhibited a noticeable decline over time. However, when irradiated mice were treated with CTI, although their weight initially decreased in the first four days, it started to recover on the fifth day and continued to show a consistent upward trend in the following days. By the 14th day, there was a significant difference in average body weight between the model group (20.05 ± 1.04 g) and the CTI group (23.17 ± 1.01 g).

We also assessed the proliferation of intestinal epithelial cells by conducting a Ki67 immunohistochemical staining assay. The number of Ki67-positive cells in the CTI group and amifostine group were significantly higher than in the model group and Cur group, indicating that CTI treatment promotes cell proliferation in irradiated mice (Figure 6F).

One of the significant damages caused by ionizing radiation to the intestine is increased intestinal permeability, which can result in intestinal dysfunction [46]. FITC–dextran, a macromolecular compound, was used to assess intestinal permeability. Both treatment groups showed significantly reduced intestinal permeability compared to the model group (*p* < 0.01, Figure 6G).

Local irradiation-induced intestinal inflammation can result in colitis, characterized by weight loss, shortened colon length, diarrhea, and bloody stools. We measured the colon lengths in male mice subjected to irradiation to assess the pathological alterations in intestinal structure. Compared to the healthy group, the colon lengths were significantly reduced in the model group, decreasing from 7.46 ± 0.42 cm to 5.13 ± 0.42 cm (Figure S7). However, the colonic shortening induced by irradiation was notably reversed by CTI treatment.

The open field test (OFT) is a commonly used approach to assess the movement ability of irradiated mice and evaluate their recovery from radiation-induced injury. Compared to the model group, both the CTI and amifostine groups showed significant reductions in movement initiation time and increases in average velocity and total distance covered (Figure S8). All these results demonstrated the effective radioprotective capability of CTI in mitigating acute injury in the small intestine. Furthermore, the protective efficiency of CTI was superior to that of natural Cur.

### 3.9. CTI Reduces the Expression of Inflammatory Cytokines and Improves Redox-Related Indicators

We further determined the antioxidation effect and anti-inflammatory activities of CTI in irradiated mice. Cytokines are crucial regulators of the immune responses of the host during infection, inflammation, and trauma. Cytokines like TNF-*α*, IL-6 and IL-1*β* play pivotal roles in the progression of inflammation. TNF-*α*, IL-6 and IL-1*β* in the model group significantly increased compared to the healthy group (*p* < 0.001, Figure 6H–J), suggesting that radiation induced the occurrence of enteritis. Conversely, the administration of CTI or amifostine led to a significant reduction in inflammatory cytokines, with levels approaching those of the healthy group. Moreover, CTI exhibited a stronger inflammatory inhibition effect than that of natural Cur, although no statistical difference was found between them.

SOD exhibited robust ability to scavenge oxygen radicals and played a crucial role in the breakdown of potentially harmful oxygen molecules [47]. MDA, a prominent lipid peroxide, possesses the capability to initiate a chain reaction of radicals [48]. In this study, the SOD levels in the model group showed a significant decrease, while the MDA levels exhibited a sharp increase compared to the healthy group (*p* < 0.001, Figure S9). In contrast, CTI treatment resulted in an increase in SOD levels and a decrease in MDA levels, indicating the effectiveness of CTI in regulating oxidative stress levels in radiation-induced enteritis.

### 3.10. CTI Maintains the Diversity of Gut Microbiota

**Figure 7:**
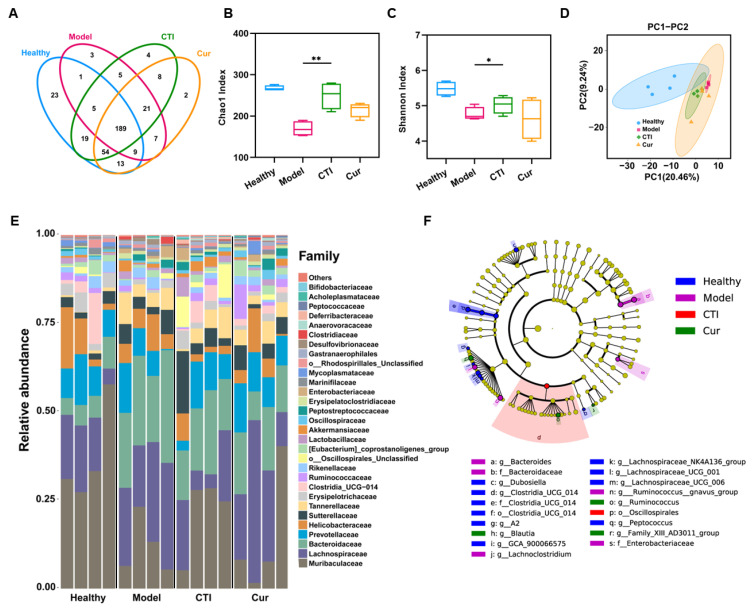
16S rDNA sequencing analysis of the gut microbiota of the various groups. (**A**) Venn diagram. (**B**) Chao 1 index. (**C**) Shannon index (vs. the model group, * *p* < 0.05, ** *p* < 0.01, n = 4). (**D**) PCA diagram. (**E**) Change in abundance and diversity of gut microbiota at the family level (n = 4). (**F**) Cladogram of the strains of identified bacteria. The color depth of each dot is proportional to its effect.

The gut microbiota executes multiple physiological functions and occupies a pivotal role in maintaining the health and intestinal homeostasis of hosts. Therefore, we further investigated the impact of ionizing radiation and the administration of CTI on the gut microbiota. In this study, we found that CTI protected the irradiated gut microbiota well, even close to the situation of healthy mice, with operational taxonomic units (OTUs) of 303 and 313, respectively. In contrast, the OTUs in the model significantly decreased to 240 (Figure 7A). Moreover, both the Chao1 index and Shannon index demonstrated that the gut microbial communities in the model group had significantly lower abundance and diversity compared to the healthy group, while no significant differences were observed between the CTI group and the healthy group (Figure 7B,C). Principal component analysis revealed that *γ*-ray irradiation significantly altered the bacterial community composition compared to the control group. The microflora composition in the two treatment groups resembled that of the healthy group. However, the intragroup differences in intestinal flora in the CTI group were significantly smaller than those in the Cur group (Figure 7D). The results indicated that CTI was more effective than Cur in maintaining the microbiota composition, probably owing to its superior solubility and bioavailability and the prebiotic properties of Inu. Notably, radiation exposure resulted in changes in the compositions of bacterial communities at various taxonomic levels, including family and genus, across the different treatment groups (Figure 7E). The abundant taxa in different groups were analyzed using linear discriminant analysis (LDA) effect size (Figure 7F). CTI significantly increased the relative abundance of *Akkermansia*, *Blautia* and *Lachnos* in the irradiated mice compared to the other irradiated groups (Figure S10). Taken together, the administration of CTI promotes an increase in the diversity of the gut microbiota in irradiated mice and restores its functionality, thereby facilitating the recovery of intestinal function.

## 4. Conclusions

In summary, in order to improve the protective effects of Cur in radiation-induced enteritis, we designed and prepared novel ROS-responsive CTI micelles. The formation of the nanomicelles dramatically increased the solubility, stability and bioavailability of Cur. Importantly, in vitro cell assays indicated that CTI exhibits superior efficacy in eliminating ROS generated by radiation, preventing DNA damage and consequently mitigating radiation-induced cell death compared to Cur. Moreover, in vivo protective assays indicated that CTI not only ameliorated the severe radiation-induced intestinal injury but also improved redox-related indicators and reduced the expression of inflammatory cytokines. The therapeutic efficacy of CTI was almost equivalent to that of amifostine and could be a promising candidate for the clinical management of radiation-induced enteritis. Our study provides a new perspective for the treatment of radiation-induced enteritis.

## Data Availability

The original contributions presented in the study are included in the article/Supplementary Material.

## Supplementary Materials

The following supporting information can be downloaded at https://www.mdpi.com/article/10.3390/antiox13040417/s1. Figure S1: Mass spectrometry of TK-COOH; Figure S2: ^1^H NMR spectrometry of TK-COOH; Figure S3: AFM images of CTI micelles; Figure S4: Digital images of CTI micelles with varied curcumin concentrations; Figure S5: Release profiles of curcumin from CTI micelles under different conditions; Figure S6: Cell viability of IEC-6 cells under *γ*-ray with different doses; Figure S7: Colon tissue pictures (A) and length (B) on day 3 postirradiation with different treatments; Figure S8: OFT results on day 7, including (A) motion trails, (B) total distance, and (C) dead time in the various groups; Figure S9: Expression of (A) SOD and (B) MDA in the small intestine tissues in different groups; Figure S10: Relative abundance of the gut microbiota of the various groups, involving (A) *Akkermansia*, (B) *Blautia*, (C) *Lachnos*. References [19,49] are cited in the Supplementary Materials.
