# Supplementary material for: Oral Curcumin–Thioketal–Inulin Conjugate Micelles against Radiation–Induced Enteritis
Source: Antioxidants (Basel). 2024 Mar 29;13(4):417. doi: 10.3390/antiox13040417 (PMC11047665; doi:10.3390/antiox13040417)
Supplement: Supplementary file 1 [file antioxidants-13-00417-s001.zip › antioxidants-2900513-supplementary.pdf]

Supporting Information

Oral Curcumin–Thioketal–Inulin Conjugate Micelles against Radiation−Induced Enteritis

Jintao Shen[1], Wencheng Jiao[1], Bochuan Yuan[1], Hua Xie[1], Ziyuan Chen[1], Meng Wei[1], Yingbao Sun[1], Yanping Wu[1], Feng Zhang[1], Zhangyu Li[1], Xu Jin[2,3,*], Lina Du[1,*] and Yiguang Jin[1,*]

1. *Beijing Institute of Radiation Medicine, Beijing 100850, China*
2. *Department of Anesthesiology, Cancer Hospital Chinese Academy of Medical Sciences, Beijing, 100191, China*
3. *Department of Anesthesiology, Beijing Tiantan Hospital, Capital Medical University, Beijing, 100070, China.*

*** Correspondence: jxsys2020@cicams.ac.cn (X.J.); dulina@bmi.ac.cn (L.D.); jinyg@bmi.ac.cn (Y.J.)**

Additional experimental details

1. Synthesis and characterization of CTI conjugates

The synthesis process consisted of three steps: synthesis of ROS-responsive linker carboxylated thioketal, thioketal carboxylate Inu to obtain Inu-TK, and esterification between Inu-TK and Cur to get CTI conjugate.

2. Synthesis of ROS-responsive linker TK-COOH

3-Mercaptopropionic acid (10.4 g，98.2 mmol) and acetone (11.6 g 196.4 mmol) were mixed in the presence of 1 ml of trifluoroacetic acid and stirred at room temperature for 6 h [49] . The mixture was quenched in the ice bath for crystallization. The crystal was washed with hexane and cold water and vacuum-dried to obtain white sold thioketal (TK-COOH, 8.7g, yield = 70.3%).

3. Synthesis of thioketal terminated Inu

Thioketal terminated Inu (Inu-TK) was synthesized according to the previously published method with minor modifications [19]. First, TK-COOH (0.5 g, 1.82 mmol) was added to 2 mL of acetic anhydride. The mixture was stirred at room temperature for 2 h under a nitrogen atmosphere. Then, 10 mL of toluene was added and the mixture was concentrated in vacuo. The process was then repeated three times. The product was redissolved in 10 mL of DMSO, Inu (1 g), and DMAP (10 mg) were subsequently added. The reaction was stirred at room temperature for 48 h and then dialyzed against deionized water for 2 days, followed by lyophilization to obtain Inu-TK.

4. Synthesis of Cur-TK-Inu conjugates

Cur-TK-Inu (CTI) was synthesized via a DCC/DMAP-catalyzed reaction. Briefly, Inu-TK (1 g) in 10 mL of dry DMSO, DCC (0.41 g), and DMAP (0.24 g) were added and the mixture was stirred for 30 min to activate the carboxyl groups. Cur (0.73 g) was added to the above-reacted solution and reacted at room temperature for 48 h in the dark. The reaction mixture was filtered and diethyl ether was added to obtain a dark orange-colored precipitate. The obtained crude product was re-dissolved in DMSO and dialyzed by DMSO and ultrapure water for 2 and 3 days, respectively (MWCO: 3500 Da). Finally, the solution was lyophilized to obtain the CTI conjugate as a yellow powder.

5. Characterization of CTI conjugates

CTI conjugates were characterized by Fourier transform infrared spectroscopy (FTIR, Spectrum Two, PerkinElmer, USA) and nuclear magnetic resonance (^{1}H NMR, Bruker 500 MHz, Germany), respectively. The UV-Vis spectra of CTI were scanned from 200 to 800 nm. The grafting rate of Cur was determined by measuring the absorbance of the Cur and CTI solutions of 100 μg/mL at 432 nm. The XRD patterns of Inu, Cur, Inu-TK, and CTI were determined using an X-ray diffractometer (Rigaku D/MAX-2600, Japan).

6. Determination of critical micelle concentration (CMC)

The determination of the critical micelle concentration (CMC) of the CTI was performed using fluorescence spectroscopy (Spark, Tecan, Swiss) with pyrene as the fluorescent probe. In summary, 30 μL of pyrene solution (8×10^{-5} M) in acetone was

added to a set of vials and subsequently evaporated to eliminate the acetone. Aqueous solutions of CTI at concentrations ranging from 0.001 μg/mL to 1 mg/mL were added to the vials. which were subjected to a 40-minute sonication within an ultrasonic bath, facilitating the establishment of equilibrium between pyrene and the conjugate. The vials were left undisturbed overnight at room temperature. The final concentration of pyrene in each vial was 8×10^{-7} M. The emission spectra were recorded at 372 nm and 383 nm with an excitation wavelength of 330 nm. The intensity ratio (I_{372}/I_{383}) of pyrene was plotted against the logarithm of conjugate concentration. The CMC was determined by taking the cross-point when extrapolating the intensity ratios.

7 Physicochemical characterizations of CTI micelles

The hydrodynamic diameter and PDI of the CTI micelles were investigated with a Malvern Zetasizer Nano ZS (Malvern Instruments, UK) at 25 °C. The micelles morphology was observed by a transmission electron microscope (TEM, Hitachi, HT7800, Hitachi Ltd., Japan) and an atomic force microscope (AFM, Bruker, Dimension ICON, German).

8 Water solubility assessment

The water solubility assessment of CTI micelles was conducted as follows: 0.1 g of CTI micelles was dissolved in 0.5 mL of water and vigorously mixed. Subsequent to centrifugation at 21,000 rpm for 10 minutes, the supernatant was isolated and further diluted with DMSO. Subsequently, the solubility of CTI was determined by measuring the absorbance of at 432 nm.

9 stability assay

The UV-vis absorption at 432 nm was employed to assess the thermal stability (37 °C, 50 °C, and 70 °C) and pH stability (1.2, 5.0, and 7.4) of both Cur and CTI micelles. The hydrodynamic diameter and PDI of the CTI micelles under different conditions were also investigated with a Malvern Zetasizer Nano ZS (Malvern Instruments, UK) at 25 °C.

10 *In vitro* release study

A 1 mL solution of CTI micelles (1 mg/mL) was transferred to a dialysis bag with a molecular weight cutoff of 3500 Da. The dialysis bag was immersed in 50 mL of PBS (pH 6.8) containing 0.1% Tween 80. To simulate the ROS environment, a H_2O_2 solution with a final concentration of 1 mM was employed. Samples of 1 mL were withdrawn at various time intervals, and 1 mL of fresh release medium was added to maintain a constant volume. The concentration of curcumin was determined using a UV-vis spectrophotometer, and the cumulative release of curcumin was calculated based on the release duration.

Additional results

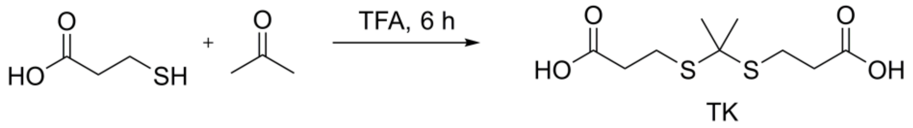

Scheme 1. Synthetic route of TK-COOH.

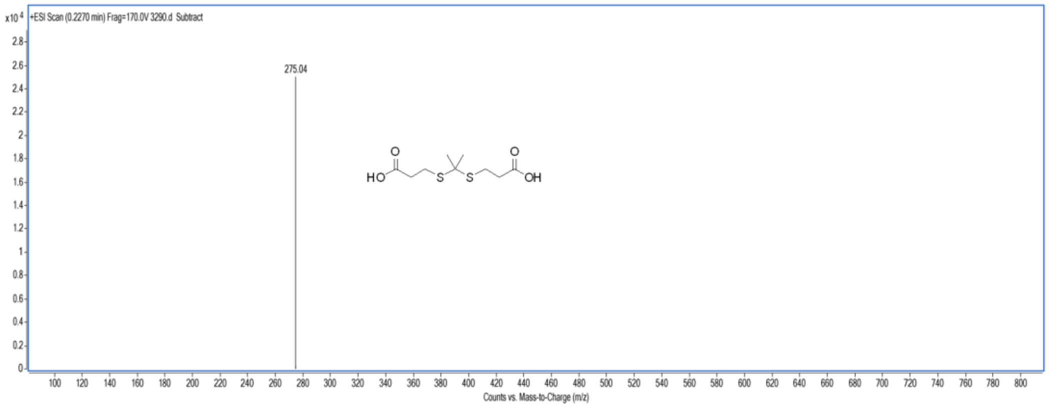

Fig. S1. Mass spectrometry of TK-COOH

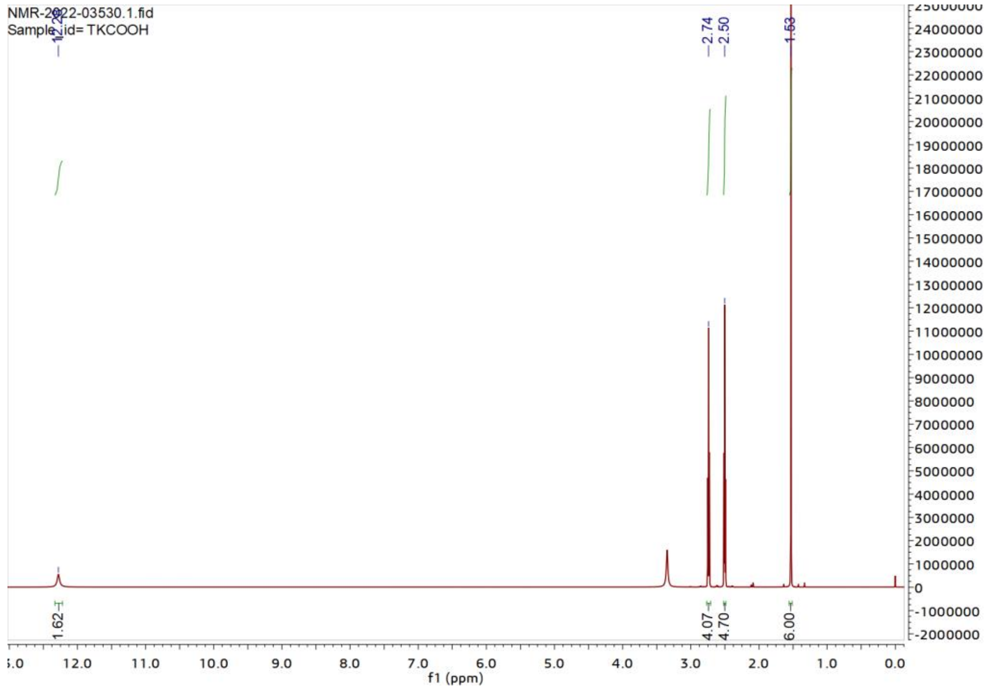

Fig. S2. ^{1}H NMR spectrometry of TK-COOH.

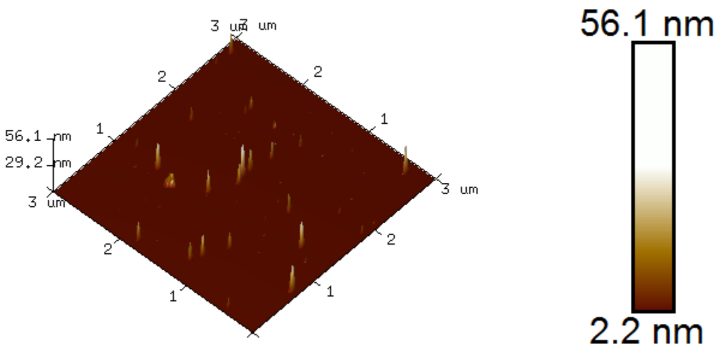

Fig. S3. AFM images of CTI micelles.

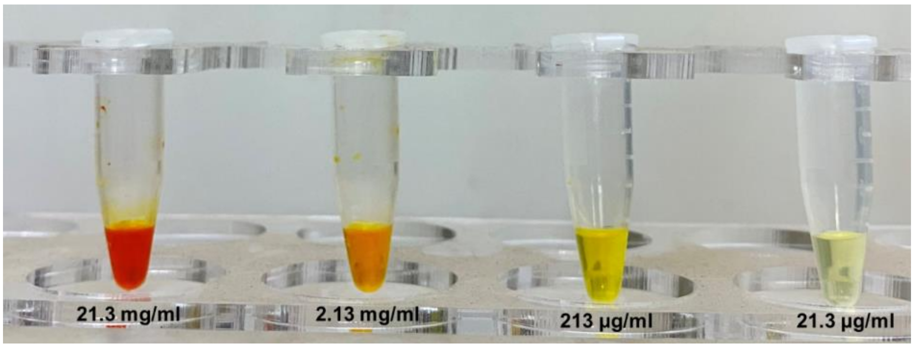

Fig. S4. Digital images of CTI micelles with varied curcumin concentrations.

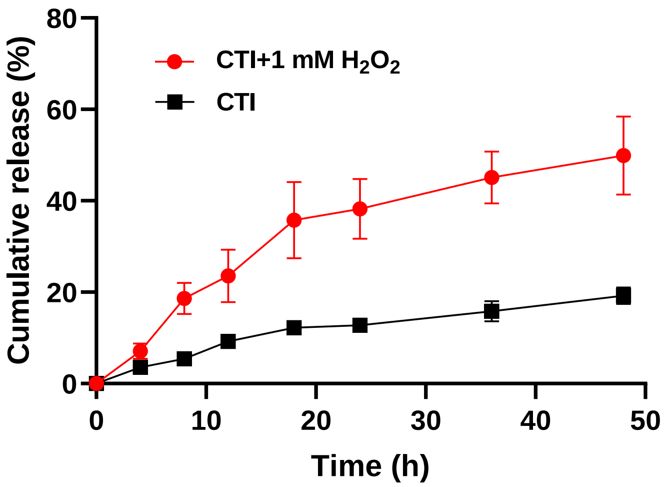

Fig. S5. Release profiles of curcumin from CTI micelles under different conditions. (n = 3)

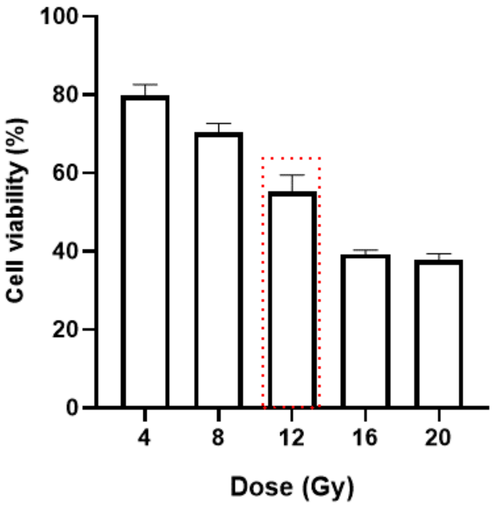

Fig. S6. Cell viability of IEC-6 cells under γ-ray with different doses.

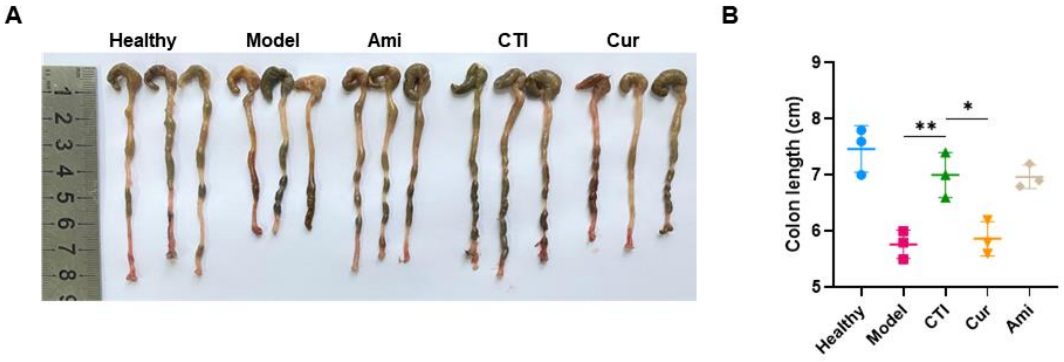

Fig. S7. Colon tissue pictures (A) and length (B) on Days 3 post-radiation with different treatment, n = 3. *P < 0.05 and **P < 0.01.

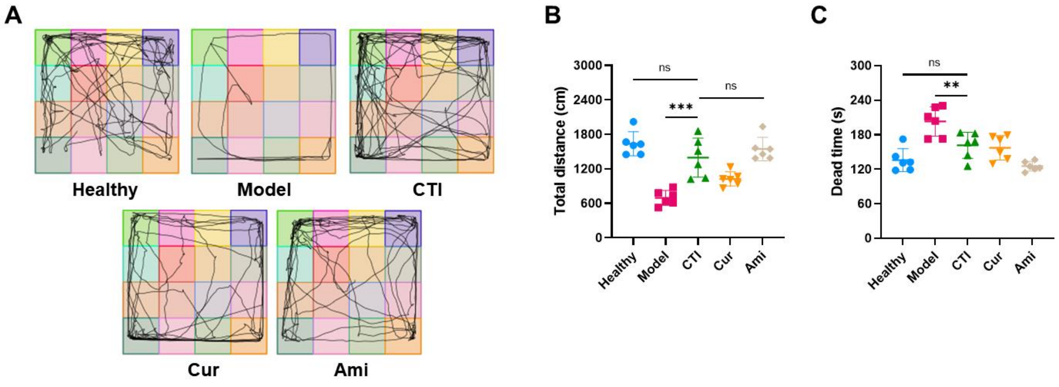

Fig. S8. OFT results on Day 7 including (A) the motion trails, (B) the total distance and (C) dead time of the various groups. n = 6. **P < 0.01 and ***P < 0.001.

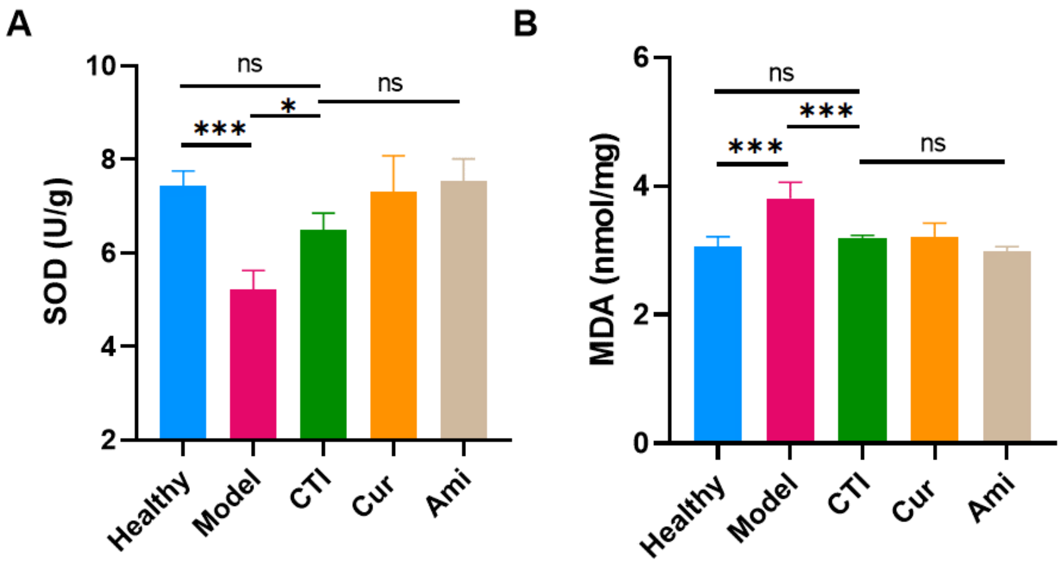

Fig. S9. Expressions of (A) SOD, (B) MDA in the small intestine tissues in different groups. Data are presented as mean ± SD, n = 4, *P < 0.05, ***P < 0.001.

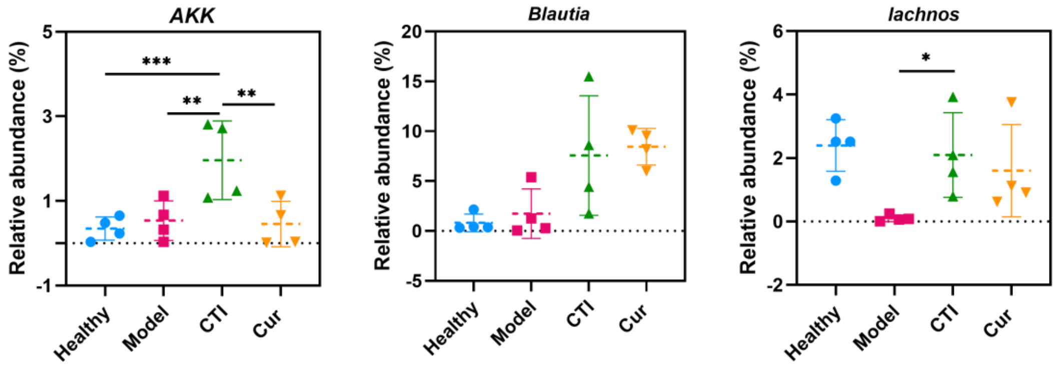

Fig. S10. Relative abundance of the gut microbiota of the various groups, involving (A) *Akkermansia*, (B) *Blautia*, (C) *Lachnos*. Data are presented as means ± SD (n = 4). *P < 0.05, **P < 0.01, ***P < 0.001.
